# Reconstruction of the coronoid process with the olecranon tip for chronic elbow dislocation in children: A rare case report and literature review

**DOI:** 10.3389/fped.2022.977866

**Published:** 2022-11-24

**Authors:** Yikun Jiang, Le Qi, Chuangang Peng, Qiwei Li, Peng Zhang, Yanbing Wang, Dankai Wu

**Affiliations:** ^1^ Department of Orthopedics, The Second Hospital of Jilin University, Changchun, China; ^2^ Department of Pediatric Orthopaedics, Shengjing Hospital of China Medical University, Shenyang, China; ^3^ Department of Radiology, The Second Hospital of Jilin University, Changchun, China

**Keywords:** coronoid process, olecranon tip, reconstruction, children, case report

## Abstract

The coronoid process of the ulna, as a key part of the elbow joint, plays an important role in maintaining elbow joint stability. Reconstruction of the coronoid process is necessary in both acute and chronic coronoid defects to restore elbow stability and avoid early joint degeneration. The olecranon tip may be a useful autologous osteochondral graft for reconstructing the same shape of the ulna coronoid process. The purpose of this report was to verify if reconstruction of the coronoid process with the olecranon tip can restore elbow stability and kinematics. Here, we report a 13-year-old boy who had undergone Kirschner-wire fixation for a left supracondylar fracture of the left humerus 9 years previously. After that, the right elbow dislocation and varus deformity gradually appeared. Imaging revealed posterolateral dislocation of the left elbow due to the absence of the coronoid process of the ulna. We reconstructed the ulnar coronoid process by intercepting the ipsilateral olecranon tip. After 22 months of follow-up, the range of motion of the left elbow joint was normal, and the cubitus varus deformity disappeared. The results of this report suggest that olecranon tip autografts are suitable to replace transverse coronoid defects. Given the patient's satisfactory clinical results, this reconstruction technique is safe and effective for the treatment of chronic elbow instability due to coronoid process defects of the ulna.

## Introduction

The coronoid process of the ulna plays a vital role in the stability of the elbow joint. As the primary osseous structure is related to the stability of the posterior elbow joint, the coronoid process not only resists the stress of the biceps, brachialis, and triceps brachii from the ulna to move backwards during flexion and extension (1–3), but also maintains the axial stability of the elbow joint and the stability of the posteromedial and posterolateral rotation (4, 5). In addition, it can prevent the occurrence of elbow varus and valgus (6, 7). In addition to these important functions related to bone structure, the coronoid process also provides attachment sites for multiple soft tissues (1). Therefore, coronoid defects of the ulna can cause not only acute and chronic joint instability, but also soft tissue instability (8, 9), which leads to posterior or recurrent dislocations of the elbow followed by rapid degeneration to posttraumatic arthritis (5, 10, 11). This shows that the coronoid process of the ulna is the main stabilizer of the elbow, and without proper treatment, it often leads to adverse outcomes (10).

In general, open reduction and internal fixation with the lateral collateral ligament and possible medial collateral ligament repair are recommended for coronoid fracture (12). However, severe comminution coronoid fractures or old coronoid defects are difficult to repair directly, and coronoid reconstruction is required to restore elbow stability (1, 13). Old coronoid process defects cannot be repaired with residual bone tissue due to bone resorption at the fracture site, resulting in elbow dislocation, traumatic arthritis, residual cubital varus deformity, and inability to perform open reduction and internal fixation of the coronoid process (5). Therefore, coronoid reconstruction or replacement is required to restore elbow stability (14).

An ideal reconstruction material should have an articular cartilage surface that matches the elbow and a radius of curvature similar to that of the natural intact coronoid process to achieve a high healing success rate (1). Therefore, we selected the ipsilateral olecranon tip as the reconstruction material. The tip of the olecranon is an intra-articular structure covered by articular cartilage, providing the advantage of an autogenous osteochondral graft that is anatomically similar to the coronoid process (10). Moreover, appropriate removal of the olecranon tip has only a slight effect on joint stability (15). In addition, the olecranon is located adjacent to the surgical site, thus reducing concerns regarding donor site morbidity at different sites (16).

In previous studies, only Moritomo et al. (17) described two adult patients who underwent reconstruction of the coronoid process using the ipsilateral olecranon tip. However, detailed clinical parameters and osteotomy procedures for coronoid and olecranon donors have not been provided. Additionally, there are some biomechanical studies on olecranon reconstruction of coronoid processes *in vitro*, but none have been proven clinically (10, 12, 18, 19). Our case report provides detailed evidence that olecranon tip reconstruction of an ulnar coronoid defect shows good long-term healing results in children.

## Case presentation

### Chief complaints and physical examinations

A 13-year-old boy had a supracondylar fracture of the left humerus due to trauma 9 years ago, and underwent Kirschner-wire internal fixation in another hospital. As detailed imaging data have been lost, the patient's parents are unable to provide us. After that, the right elbow dislocation and varus deformity gradually appeared. Physical examination revealed a 10 cm longitudinal scar on the left elbow. Obvious osterior dislocation of the left elbow was observed in the extension position, with an obvious varus deformity. Automatic reduction was observed in the flexion position. See Supplementary Material 1.

### Imaging examinations and final diagnosis

**Figure 1:**
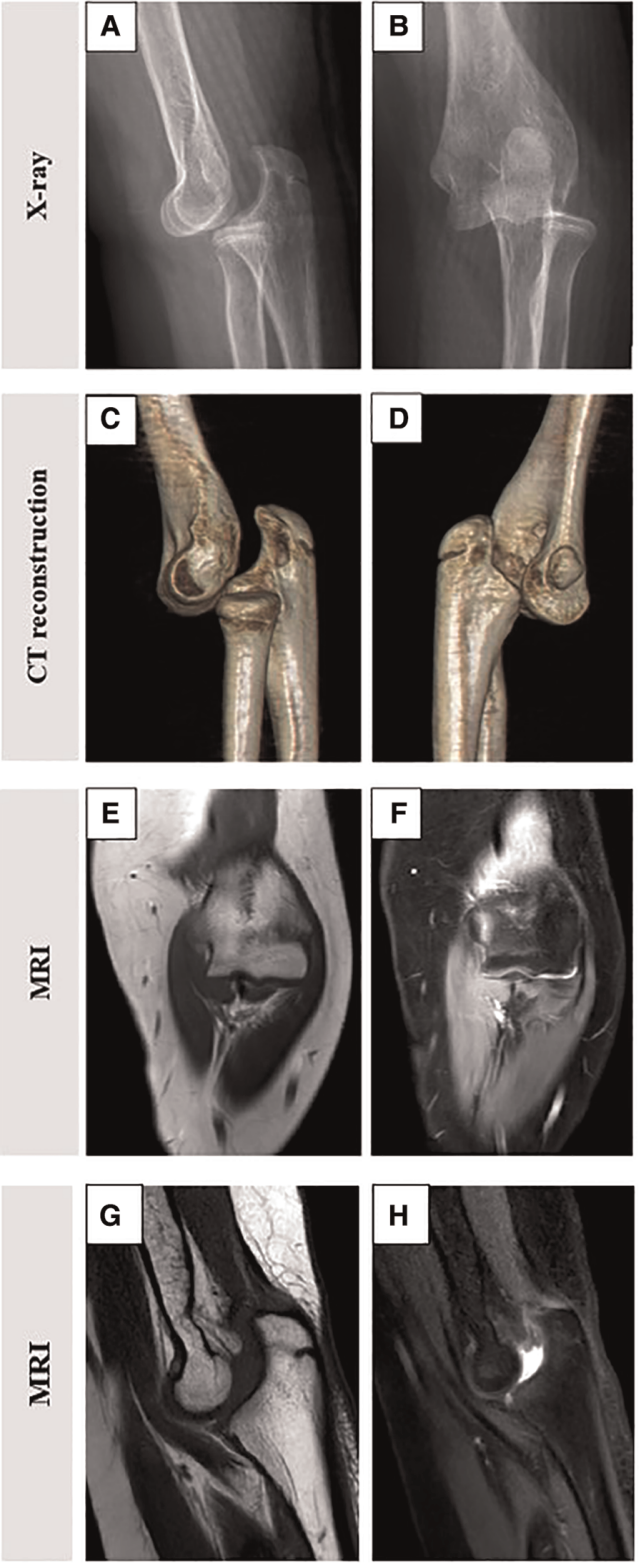
Preoperative imaging showing posterolateral dislocation of the left elbow. (**A,B**) Radiographs show anteriorly inferior slippage of the distal humerus without fracture; (**C,D**) Three-dimensional computed tomography shows a transverse defect of the coronoid process of the ulna, (**E,F**) The MRI of left elbow shows no injury of medial collateral ligament and lateral collateral ligament. (**G,H**) The MRI of left elbow shows posterolateral dislocation.

Radiography and three-dimensional computed tomography (CT) revealed partial absence of the coronoid process of the left ulna. The left distal humerus was displaced anteriorly and downward, and there was no bone fracture in the left elbow. The magnetic resonance imaging (MRI) of the left elbow showed posterolateral dislocation of the left elbow and there was no injury in medial collateral ligament and lateral collateral ligament, as shown in Figure 1. Combined with the patient's physical and imaging findings, we made a final diagnosis of posterior dislocation and varus deformity of the left elbow.

### Treatment

Based on the patient's physical examination and imaging findings, an ipsilateral olecranon osteotomy for reconstruction of the coronoid process of the ulna was performed to treat elbow dislocation. The skin, subcutaneous tissue, and myofascial membrane were sequentially cut by making an anterior s-shaped incision of length 12.0 cm on the left elbow. The median nerve was exposed on the medial side of the biceps tendon and the left elbow joint capsule was opened. After full exposure, the left elbow joint coronoid process cartilage and a part of the bone were found missing. The length of the longitudinal surgical incision at the olecranon at the back of the left elbow was approximately 4 cm to fully expose the olecranon. The left olecranon tip of the left ulna, approximately 1.5 cm × 1 cm in size, was taken. After repair, it was implanted into the coronoid process defect. Two 1.0 g wires were used for temporary fixation, and the bone and cartilage of the ulnar coronoid process were well-reconstructed. Then, a suitable T-shaped plate was placed, and the locking screws were screwed for fixation. C-arm fluoroscopy revealed that the internal fixator was in a good position. Reconstruction of the coronoid process was stable, and the elbow joint was not dislocated. Finally, the two 1.0 g wires were removed. The anterior and posterior surgical incisions of the left elbow were sutured layer-by-layer, and the left elbow was externally fixed with plaster. The total operative time was 3 h. After 6 weeks, the cast was removed, and elbow movement gradually restored.

### Outcome and follow-up

**Figure 2:**
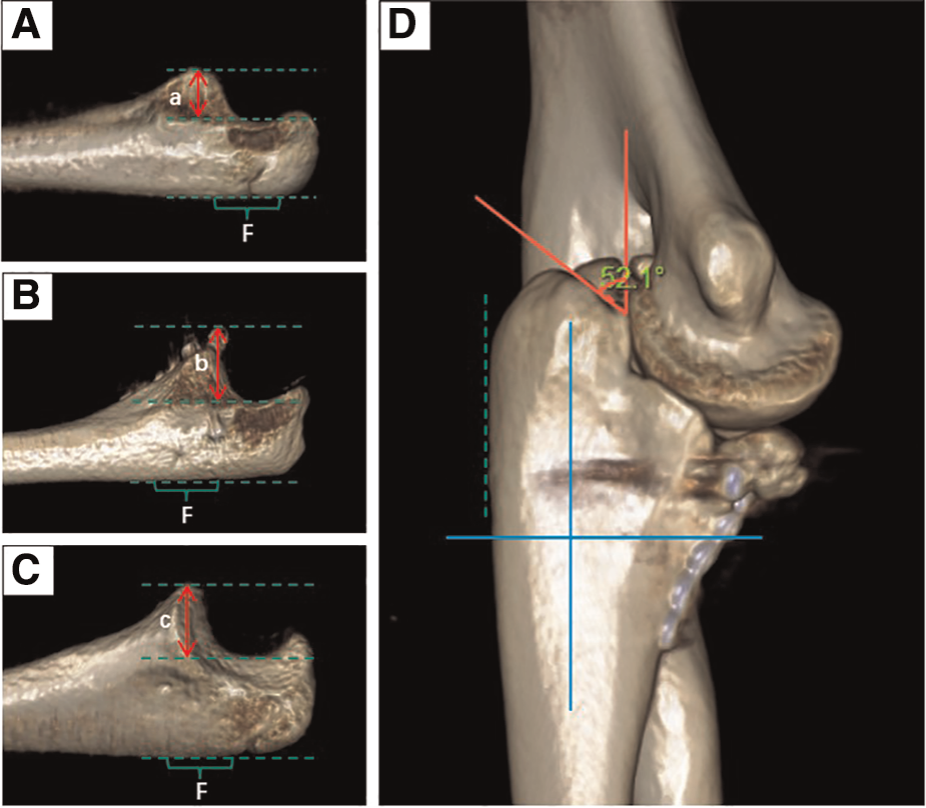
Parameters of the coronoid process of the ulna and the tip of the olecranon. (**A**) represents the height of the coronoid process of the ipsilateral ulna preoperatively; (**B**) represents the height of the coronoid process of the ipsilateral ulna postoperatively; (**C**) represents the height of the unaffected coronoid process; (**D**) shows that the osteotomy angle of the olecranon is about 52.1°. (*a*/*c* = 0.875, *b*/*c* = 1.2). *F* is a flat spot in the proximal ulna. The three dashed lines represent the highest point of the coronoid process of the ulna, the lowest point of the sigmoid notch, and the level of the flat spot (green lines). The 0° angle runs parallel to the flat spot of the corresponding olecranon, and is defined by the constructed coordinate system (blue lines).

**Figure 3:**
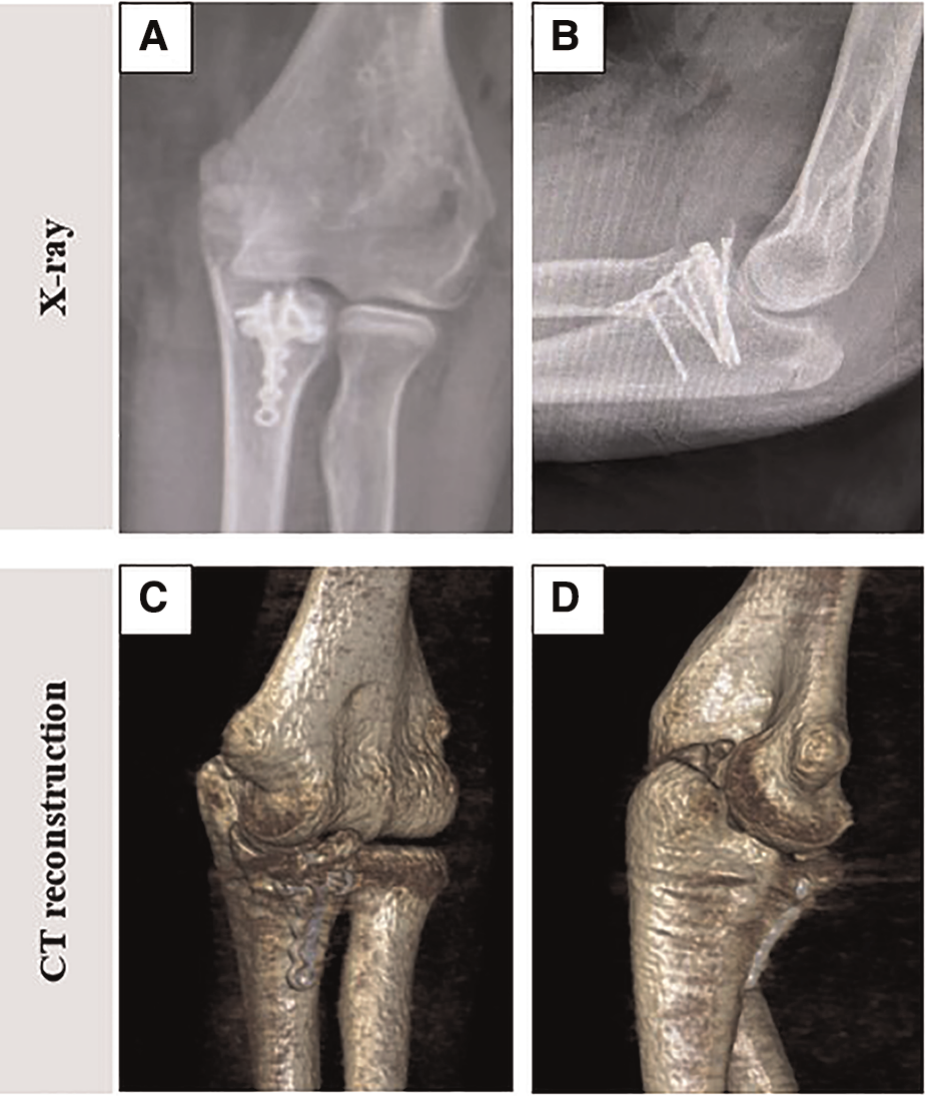
Postoperative imaging showed Normal recovery of the left elbow dislocation. (**A,B**) Radiographs show that the internal fixation device is in good position; (**C,D**) Three-dimensional computed tomography showed no arthritic or graft absorption of the elbow.

**Figure 4:**
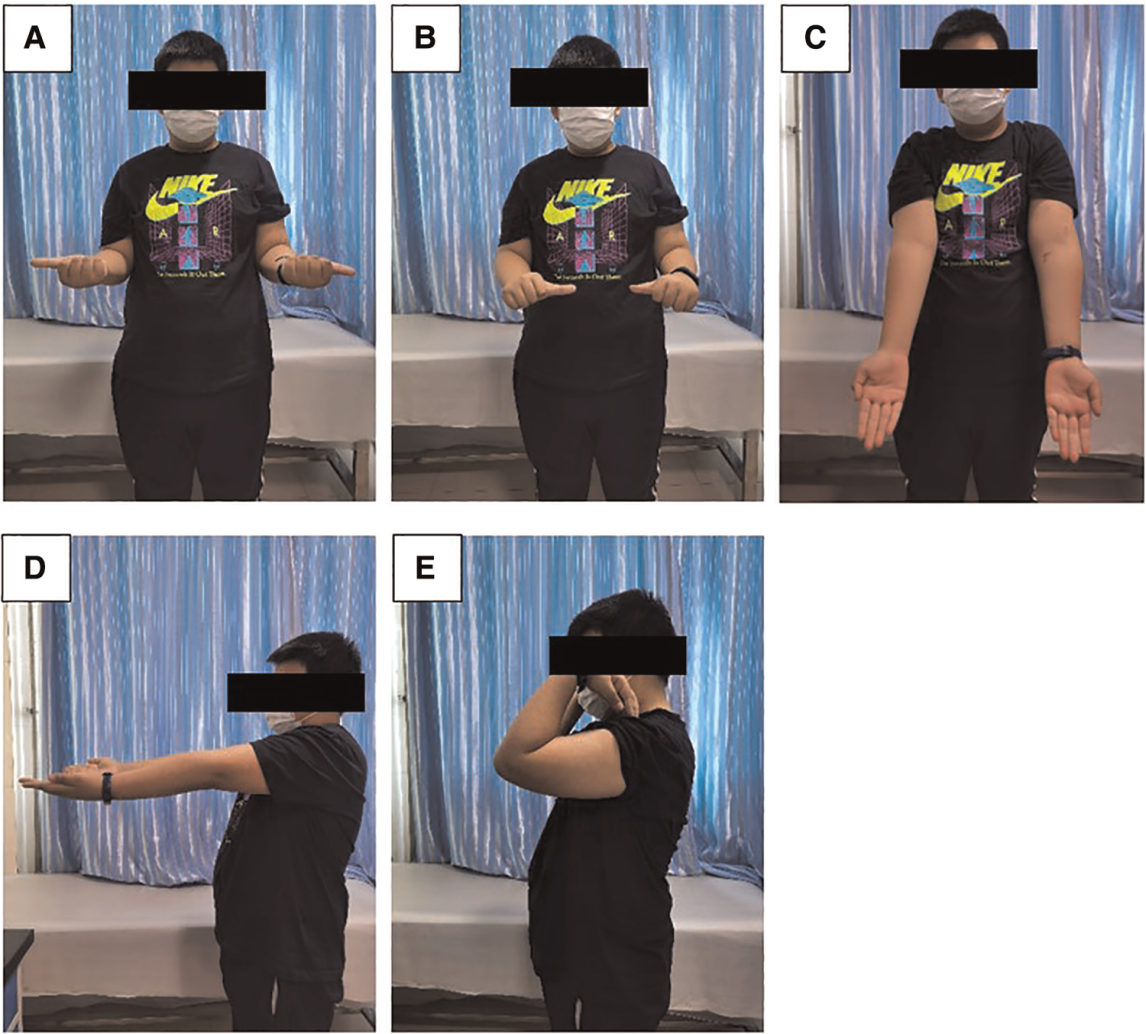
After 22 months of follow-up, the functional range of motion of the patient's upper limbs was symmetrical. (**A**) External rotation; (**B**) Internal rotation; (**C**) Extension (front view); (**D**) Extension (side view); **(E**) Flexion (side view).

The height of the coronoid process of the healthy and affected sides and the osteotomy angle of the olecranon tip were measured postoperatively according to previously used *in vitro* biomechanical analysis methods (10, 12). Measurements showed a preoperative height defect of 12.5% of the coronoid process, and the height of the reconstructed coronoid was 1.2 times that of the unimpaired side (Figures 2A–C). We also measured the angle of the olecranon tip, which was 52.1° (Figures 2D). At the 10th month follow-up, radiography and three-dimensional CT results of the left elbow showed that the internal fixation device was in a good position without complications, such as displacement and fracture, and there were no manifestations of osteoarthritis changes or graft absorption, as shown in Figure 3. After 22 months of follow-up, the patient had a symmetrical range of motion in both elbow joints with no residual dysfunction, as shown in Figure 4.

## Discussion

The first step in the treatment of elbow instability with coronoid process fracture is to determine whether elbow instability is caused by the injury. If the coronoid process is determined to be the cause, caution should be exercised in the treatment of elbow instability with reconstruction. According to previous literature, the indications for coronoid reconstruction surgery can be summarized as follows (13, 20–23): (1) Regan–Morrey or O'Driscoll type III fresh comminuted fractures, (2) old coronoid fractures, and (3) elbow instability after surgical or non-surgical treatment.

Previous studies have shown that autografts of the radial head, distal clavicle, costal cartilage, iliac crest cortex, and fibula can be used to reconstruct the coronal processes of the ulna. However, different materials have diverse advantages, disadvantages, or limitations (13). For example, complications such as ectopic ossification and unstable elbow joints can easily occur after radial head surgery (24). Poor homogeneity of the iliac crest cortical bone and lack of cartilage on the surface of the iliac crest increases the incidence of postoperative arthritis (25). Autografts of the distal clavicle may not be able to completely reconstruct the anterolateral and anteromedial coronal processes, and are not suitable for the reconstruction of large defects in these specific areas (14). There is also a degree of donor-site morbidity, with unpredictable outcomes. With the development of prosthetic materials and techniques, there has been progress in the application of prostheses for coronoid reconstruction. In 2017, Bellato and O'Driscoll (21) performed coronoid reconstruction in three cases using a non-anatomical metal prosthesis for the first time. After long-term follow-up, the range of motion of the elbow joint improved to varying degrees, and the position of the coronoid prosthesis remained fixed. However, they also acknowledge the disadvantage of using prostheses to reconstruct the coronoid process, as anatomical consistency requires more complex designs and a potentially wider range of size and shape choices. It is important to determine the most important aspects of the coronoid shape to mimic. However, they can be too expensive, making surgery unaffordable for most patients.

To date, the most studied reconstruction material has been the tip of the olecranon. In clinical application, Moritomo et al. first reported two cases using the ipsilateral olecranon tip to reconstruct the coronoid for the treatment of elbow dislocation, but both were applicable to adults, and did not provide detailed measurement parameters of the coronoid and olecranon (17). In an *in vitro* mechanical analysis, Kataoka et al. (12) used *in vitro* biomechanical studies in a cadaveric model to determine whether reconstruction of the coronoid process using the tip of the ipsilateral olecranon would restore the baseline kinematics of the coronoid-deficient elbow. They demonstrated that 40% of transverse coronoid defects caused major changes in the kinematics of the elbow in varus orientation. Simultaneously, a part of the olecranon tip was intercepted for reconstruction, and the distance from the olecranon tip was equal to 40% of the coronoid process height. The results show that this technique can effectively restore the range of motion of the elbow from 20° to 120°, which may be beneficial for patients with elbow instability due to non-reconstructive comminuted coronoid fractures or non-unions. In addition, they demonstrated that resection of no more than 20% to 25% of the olecranon tip did not result in substantial changes in elbow kinematics. Therefore, they believe that the olecranon tip is the most suitable material for coronoid reconstruction. Bell et al. (26) performed a biomechanical study using fresh frozen elbow samples to assess the effect of olecranon on elbow stability. They found that 50% resection of the olecranon had no significant effect on elbow stability, including varus, valgus, and rotation, which was a conclusion consistent with that obtained by An et al. (27).

In 2015, Ramirez et al. (15) used the tip of the olecranon to reconstruct the coronoid process of the ulna. In *in vitro* mechanical analysis, it was found that not only did the olecranon graft provide a continuous osteochondral articular surface in all specimens, but that the bone remodeling prior to loading did not impede the range of motion of the elbow in any specimen. No significant graft displacement or rotation was observed during testing. Statistical analysis before and after reconstruction revealed that at 15°, 45°, 75°, 90°, and 105° elbow flexion, autogenous bone olecranon tip transfer restored the stability of the back of the elbow to a level that was not significantly different from that of the intact elbow. However, they acknowledge that this biomechanical study was based only on an isolated coronoid fracture model, and did not replicate the dreaded triad injury with collateral ligament and radial head injuries. Kataoka (19) analyzed the 3D morphological features of three autologous osteochondral grafts for coronoid reconstruction: the tip of the olecranon, lateral radial head, and proximal radial head. The results showed that the coverage of the olecranon graft was significantly higher than that of the lateral and proximal radial head grafts, probably because the olecranon and coronoid tips are convex in the coronal plane. Olecranon grafts are best suited for coronoid defects, including the tips. In addition, reconstruction of 50% of the coronoid height with an olecranon graft does not use enough ulnar articular surface, nor does it raise concerns about the severe instability of the elbow.

To further determine the shape matching between the ipsilateral and contralateral olecranon tips for graft selection, as well as determine the effect of osteotomy angle on reconstruction, Wegman (10) designed six angles ranging from 10° to 60° in a coronoid process model with a 40% height defect. The results showed that the olecranon tip showed a better shape match to the natural coronoid process when osteotomy was performed at a higher angle (especially at 50°). Simultaneously, the shape match of the contralateral olecranon tip was significantly greater than that of the ipsilateral olecranon tip graft.

Besides, the related ligamentous structures of the elbow joint play an indispensable and important role in its stabilization system. The maintenance of lateral stability of the elbow joint mainly depends on the collateral ligaments, including medial collateral ligament complex (MCLC) and lateral collateral ligament complex (LCLC) (28). The lateral ulnar collateral ligament (LUCL) is considered to be the portion of the lateral collateral ligament playing the most important stabilizing role, can effectively resist the posterolateral rotation of the elbow joint (2). In our case report, none of the imaging findings showed damage to the ligaments or joint capsule, only the defect of the coronoid process of the ulna resulted in the patient's posterolateral dislocation of the elbow.

Despite the positive clinical results in this case report, there are still many limitations. Including the following aspects: (1) more clinical cases are needed to confirm the effectiveness of this procedure; and (2) the patients had chronic ulnar coronoid process defects, which did not involve the medial and lateral collateral ligaments. Therefore, we could not evaluate the therapeutic effects of this method for ligaments. In summary, a multicenter trial with a large sample size should be established.

In conclusion, a method of olecranon tip osteotomy for the reconstruction of the coronoid process of the ulna for chronic dislocation of the elbow joint has been reported. There was no iatrogenic vascular or nerve injury, and the elbow was restored to its normal range of motion. Radiographic results showed good elbow position, no graft dislocation, no osteoarthritic changes, and no radiological evidence of graft absorption. Therefore, this is a safe and effective method for treating chronic dislocation of the elbow caused by a defect in the coronoid process of the ulna in children.

## Data Availability

The original contributions presented in the study are included in the article/Supplementary Material, further inquiries can be directed to the corresponding author/s.
