# Supplementary figures and images for: Reconstruction of the coronoid process with the olecranon tip for chronic elbow dislocation in children: A rare case report and literature review
Source: Front Pediatr. 2022 Nov 24;10:977866. doi: 10.3389/fped.2022.977866 (PMC9730028; doi:10.3389/fped.2022.977866)

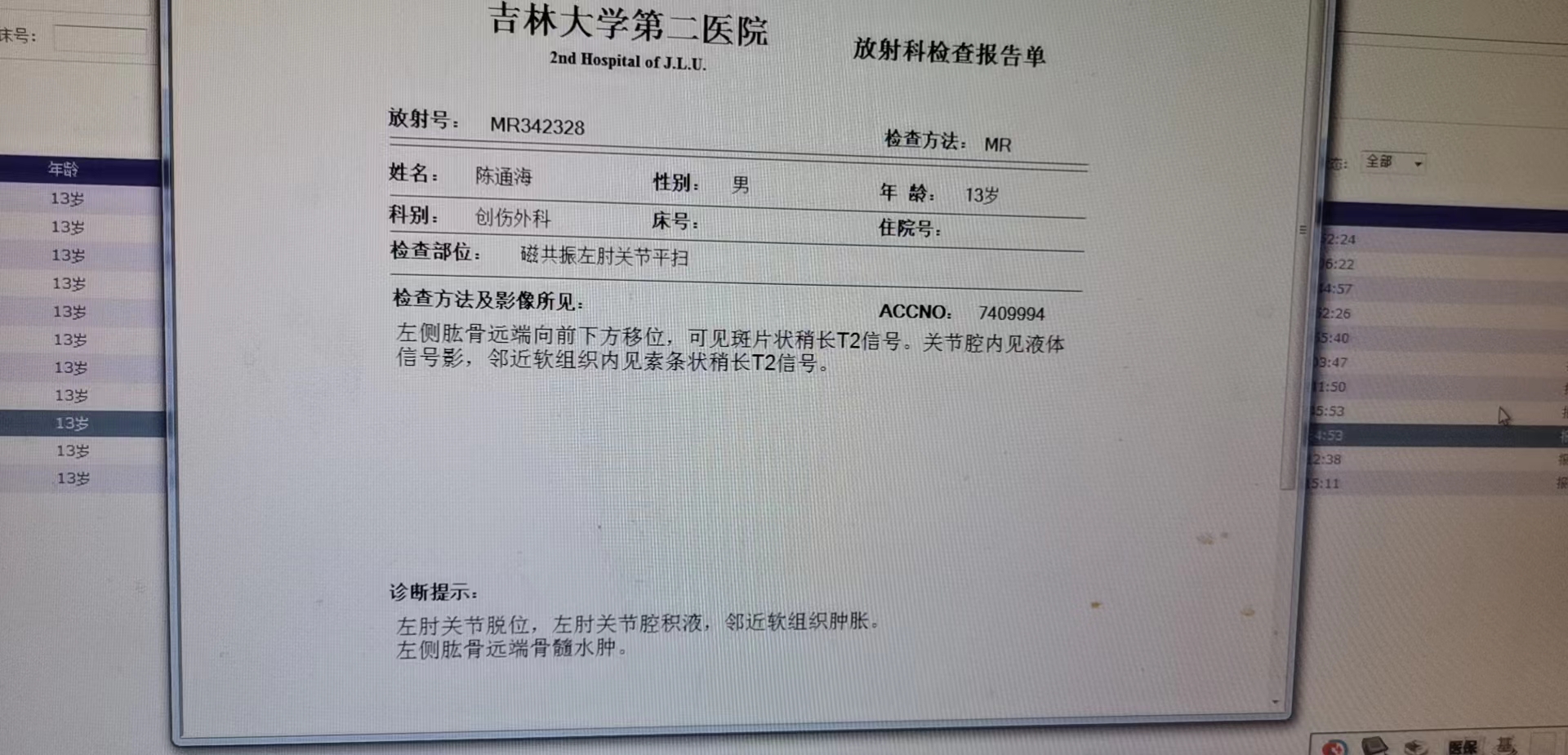

Supplement: Supplementary file 1 [file Image1.jpeg]

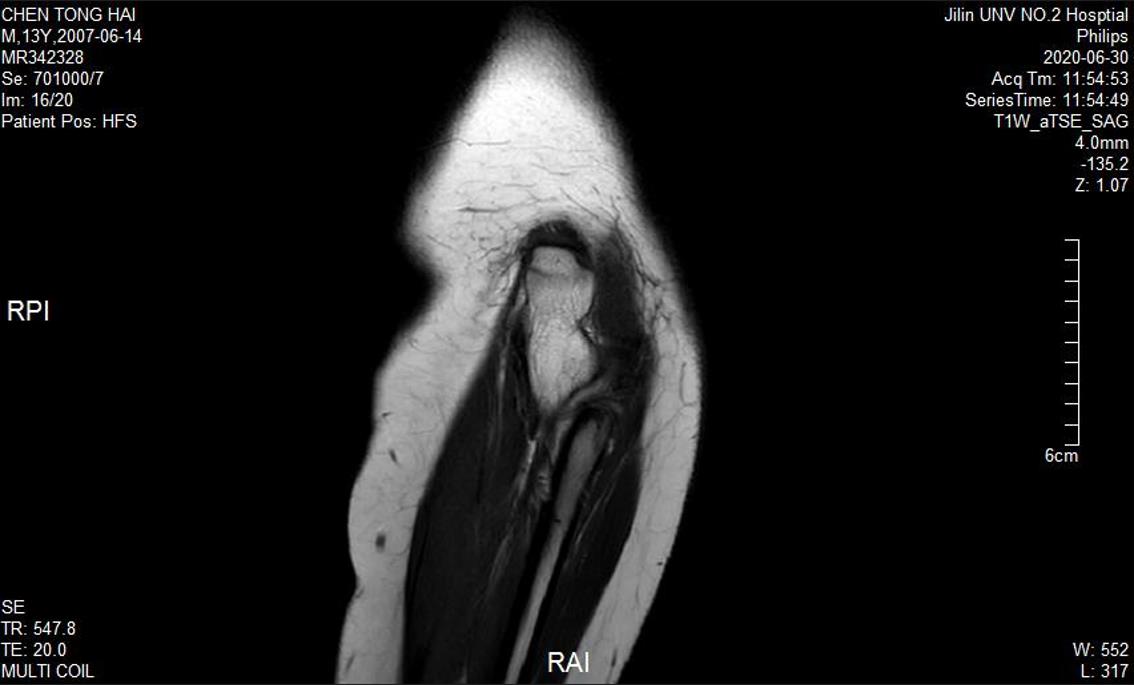

Supplement: Supplementary file 2 [file Image2.jpeg]

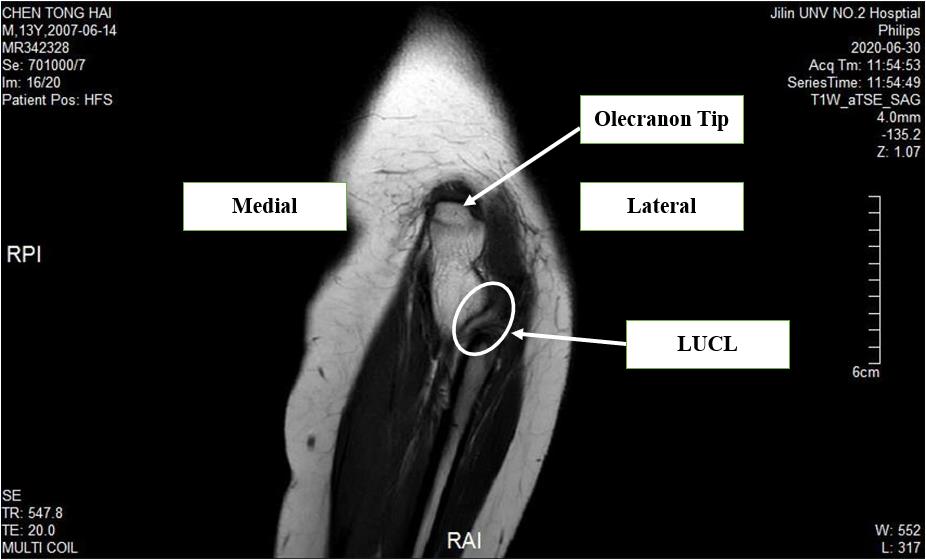

Supplement: Supplementary file 3 [file Image3.jpeg]
